# Matrix metalloproteinase‐9 inhibition or deletion attenuates portal hypertension in rodents

**DOI:** 10.1111/jcmm.16940

**Published:** 2021-10-14

**Authors:** Hui‐Chun Huang, Hsin‐Ling Ho, Ching‐Chih Chang, Chiao‐Lin Chuang, Chon Kit Pun, Fa‐Yauh Lee, Yi‐Hsiang Huang, Ming‐Chih Hou, Shao‐Jung Hsu

**Affiliations:** ^1^ Faculty of Medicine National Yang‐Ming University School of Medicine Taipei Taiwan; ^2^ Faculty of Medicine National Yang Ming Chiao Tung University Taipei Taiwan; ^3^ Division of Gastroenterology and Hepatology Department of Medicine Taipei Veterans General Hospital Taipei Taiwan; ^4^ Division of General Medicine Department of Medicine Taipei Veterans General Hospital Taipei Taiwan; ^5^ Division of Gastroenterology and Hepatology Department of Medicine Lo‐Hsu Medical Foundation Lotung Poh‐Ai Hospital Yilan Taiwan

**Keywords:** angiogenesis, liver cirrhosis, metalloproteinase, portal hypertension, portosystemic collaterals

## Abstract

Liver cirrhosis and portal hypertension are accompanied by hyperdynamic circulation, angiogenesis and portosystemic collaterals. Matrix metalloproteinases (MMPs) participate in fibrogenesis and angiogenesis, however, whether they can be targeted in cirrhosis treatment is unclear. Therefore, we performed three series of experiments to investigate this issue. Liver cirrhosis was induced by common bile duct ligation (BDL) in Sprague‐Dawley rats. Sham‐operated rats served as controls. Rats were randomly allocated to receive vehicle, minocycline (a nonselective MMP inhibitor) or SB‐3CT (MMP‐2 and −9 inhibitor) for 28 days in the first and second series, respectively. MMP‐9 knockout mice were used in the third series. The results showed that minocycline ameliorated portal hypertension, hemodynamic abnormalities, reduced collateral shunting, mesenteric vascular density, plasma VEGF level and alleviated liver fibrosis. SB‐3CT attenuated portal hypertension, hemodynamic derangements, reduced shunting, mesenteric vascular density, mesenteric VEGF protein expression, and liver fibrosis. Knockout BDL mice had significantly alleviated portal hypertension, liver fibrosis, liver α‐SMA and mesenteric eNOS protein expressions compared to wild‐type BDL mice. Liver SMAD2 phosphorylation was down‐regulated in all series with MMP inhibition or knock‐out. In conclusion, MMP‐9 inhibition or deletion ameliorated the severity of cirrhosis, portal hypertension, and associated derangements. MMP‐9 may be targeted in the treatment of liver cirrhosis.

## 1 INTRODUCTION

Liver injuries are usually followed by fibrogenesis and finally cirrhosis. Intrahepatic resistance is elevated in liver cirrhosis due to collagen fiber deposition and enhanced intrahepatic vasoconstriction.^1^ Furthermore, increased splanchnic and peripheral vasodilatory substances such as nitric oxide elevate portal inflow and pressure, leading to hyperdynamic circulation characterized by decreased peripheral vascular resistance, tachycardia and increased cardiac output. Splanchnic angiogenesis also participates in the development of splanchnic hyperemia and collaterals, in which vascular endothelial growth factor (VEGF) plays a crucial role.^2^ finally leading to portal hypertension.^3^ Portosystemic collaterals form due to obstruction of portal venous blood flow to the liver, which can then lead to fatal complications such as gastroesophageal varices haemorrhage. Considering the pathophysiological mechanisms, strategies for controlling portal hypertension‐related complications include alleviating mesenteric angiogenesis, liver fibrosis, collaterals, and correcting abnormal vasoresponsiveness.

Matrix metalloproteinases (MMPs) are essential for extracellular matrix remodelling and angiogenesis. The synergistic actions of VEGF and MMPs have been reported, in which VEGF increases the release of MMPs and decreases the release of tissue inhibitor of metalloproteinases (TIMPs). Furthermore, MMPs induce the release and angiogenic activity of VEGF^4^ are also involved in VEGF‐VEGF receptor interactions.^5^ Among the MMPs, MMP‐9 has been shown to play an essential role during the acquisition of an angiogenic phenotype.^6^ MMP‐9 is predominantly secreted by inflammatory cells such as tumor‐associated macrophages, which are found at the sites of pathological angiogenesis and are important for tumor angiogenesis.^7^

MMPs are particularly important for the development of liver fibrosis as they degrade type IV collagen (basal membrane).^8^ However, not all MMPs inhibit hepatic fibrogenesis. MMP‐9 activates transforming growth factor (TGF‐β), a cytokine that enhances fibrogenesis,^9^ and it is also upregulated in liver fibrosis.^10^ In bile duct ligation (BDL) mice, general inhibitors of MMPs have been shown to attenuate hepatic fibrosis.^11^ More specifically, MMP‐9‐deficient mice have been reported to exhibit moderate protection against early fibrosis and proteolytic inactive MMP‐9 mutants, acting as TIMP‐1 antagonists *in vitro* and inhibiting CCl_4_‐induced liver fibrosis in mice.^12^, ^13^ Nevertheless, investigations focused on portal hypertension‐related derangements are lacking.

Considering the remarkable influences of MMP‐9 on angiogenesis and fibrogenesis, we hypothesized that MMP‐9 inhibition or deletion may attenuate portal hypertension and the associated derangements. To test this hypothesis, we firstly investigated the effects of minocycline, a nonselective MMP inhibitor, because of its well‐established safety profile. We then focused on MMP‐2 and MMP‐9 inhibition based on the literature review of potentially treatable targets,^9^, ^10^, ^12^, ^13^ and finally confirmed the results with MMP‐9 knockout mice.

## 2 MATERIALS AND METHODS

### 2.1 Animal model: Common bile duct ligation (BDL)

Male Sprague‐Dawley rats weighing 240–270 g at the time of surgery were used. The rats were caged at 24℃, with a 12‐h light‐dark cycle and free access to food and water until the time of the experiments. Secondary biliary cirrhosis was induced with BDL as previously described.^14^ A high yield of secondary biliary cirrhosis was noted 4 weeks after BDL.^15^ To avoid a coagulation defect, the BDL rats received a weekly vitamin K injection (50 μg/kg intramuscularly).^15^ This study was approved by Taipei Veterans General Hospital Animal Committee (IACUC 2016–218). All animals received humane care according to the criteria outlined in the ‘Guide for the Care and Use of Laboratory Animals, 8th edition, 2011’ published by the US National Research Council.

### 2.2 Study design

Sham (surgical control) and BDL rats were randomly allocated to receive: (1) minocycline (120 mg/kg/day, oral gavage, this dose was chosen according to previous mouse models^16^, ^17^, ^18^) or vehicle (distilled water); (2) SB‐3CT (0.16 mg/kg/day, i.p.) or vehicle (phosphate‐buffered solution; PBS) for 4 weeks from the first day after surgery. This dosage of SB‐3CT was chosen because of the significant inhibitory effects on MMP‐2 and MMP‐9‐dependent ulcer healing demonstrated in a previous study.^19^ On the 29th day, three parallel series of experiments were performed. Male MMP‐9 knockout mice (MMP‐9 KO, MMP‐9 homozygous, MMP‐9^−/−^, Cg‐MMP9^tm1Tvu/J^; Jackson Laboratory) and their corresponding wild‐type (WT) littermates (C57BL/6) (WT) received BDL or sham operations. The experiments were performed on the 29th day after surgery.

### 2.3 Measurement of systemic and portal haemodynamics

The right femoral arteries of the rats were cannulated with a PE‐50 catheter connected to a Spectramed DTX transducer (Spectramed Inc.). Continuous recordings of mean arterial pressure (MAP), heart rate and portal pressure (PP) were performed on a multi‐channel recorder (RS 3400, Gould Inc.). The external zero reference was at the level of the mid‐portion of the rat. The abdomen was then opened with a mid‐line incision, and a mesenteric vein was cannulated with the PE‐50 catheter connected to the Spectramed DTX transducer. The abdominal cavity was closed and the PP was recorded using the Gould RS 3400 recorder.^20^

The superior mesenteric artery (SMA) was identified at its aortic origin and a 5‐mm segment was gently dissected free from surrounding tissues. A pulsed‐Doppler flow transducer (T206 small animal blood flow meter, Transonic System Inc.) was then placed to measure SMA flow.

Cardiac output was measured by thermodilution, as previously described.^21^ Briefly, a thermistor was placed in the aortic arch just distal to the aortic valve, and a thermal indicator (100 μl of normal saline) was injected into the right atrium through a PE‐50 catheter. The aortic thermistor was connected to a Columbus Instruments Cardiotherm 500‐AC‐R (Columbus Instruments International Co.). Five thermodilution curves were obtained for each cardiac output measurement. The final cardiac output value was calculated as the mean of the results. Cardiac index (CI, ml/min/100 g body weight [BW]) was calculated as cardiac output per 100 g BW. Systemic vascular resistance (SVR, mmHg/ml/min/100 g BW) was calculated by dividing MAP by CI. SMA resistance (mmHg/ml/min/100 g BW) was calculated as (MAP‐PP)/SMA flow per 100 g BW.

### 2.4 In situ perfusion preparation

#### 2.4.1 SMA perfusion

The *in situ* perfusion technique was modified from a previously reported *in vitro* SMA perfusion technique.^22^ The abdomen was opened and an 18‐gauge Teflon cannula serving as the inlet was inserted in the SMA. Another 16‐gauge Teflon cannula serving as the outlet was inserted in the proximal end of the superior mesenteric vein. A ligature was tied over the proximal site of the insertion site to exclude the liver and collateral from perfusion. The animal was then transferred into the upper compartment of a warm chamber (37 ± 0.5℃). The temperature around the perfusion area was continuously monitored with a thermometer placed inside the mesentery and maintained at approximately 37 ± 0.5℃ with a thermostatic pad and temperature‐controlled infrared lamp. Open circuit perfusion was then started with Krebs solution (composition in mM: NaCl, 118; KCl, 4.7; KH_2_PO_4_, 1.2; MgSO_4_, 1.2; CaCl_2_, 2.5; NaHCO_3_, 25; dextrose, 11.0; pH, 7.4; 37 ± 0.5℃) via the mesenteric cannula using a roller pump (model 505S; Watson‐Marlow Limited, Falmouth, Cornwall, UK). The perfusate was equilibrated with carbogen gas (95% O_2_‐5% CO_2_) using a silastic membrane lung. The SMV cannula was opened to allow free and complete washout of the blood. Pneumothorax was created by opening slits through the diaphragm to increase resistance in pulmonary arteries and prevent the perfusate from entering left heart chambers. The SMA was then perfused with oxygenated (95% O_2_‐5% CO_2_) Krebs solution containing 3% wt/vol albumin (factor V bovine serum albumin; Sigma). The effluent of the perfused tissue was collected in a reservoir placed at the lower compartment of the warm chamber and not recirculated. To continuously monitor and record the pressure of this territory, a Spectramed DTX transducer attached to the Gould model RS 3400 recorder was connected to a side arm placed just proximal to the perfusion cannula, with the zero placed at the level of the right atrium. Because the temperature and pressure of the system stabilized within 10 min, all experiments were performed 20 min after starting perfusion at a constant flow rate of 15 ml/min. The perfusion flow rate was kept constant so that changes in perfusion pressure reflected changes in splanchnic vascular resistance. Only one concentration‐response curve was performed in each preparation. In each individual preparation, after testing the experimental agents, the contracting capability of the splanchnic vasculature was challenged with 125‐mM potassium chloride solution at the end of the experiments.

#### 2.4.2 Liver perfusion

The *in situ* perfusion system was performed as previously described with some modifications.^23^ Briefly, both jugular veins were cannulated with 16‐gauge Teflon cannulas to ensure an adequate outflow without any resistance even at the highest flow rates. Heparin (200 U/100 g body weight) was injected through one of the cannulas. The abdomen was then opened and a 16‐gauge Teflon cannula inserted into the portal vein. The hepatic artery was ligated. To exclude the collateral vasculature from perfusion, a second loose ligature around the distal portal vein was tied. Open circuit perfusion was then started with oxygenated Krebs solution in a warm chamber. Both the jugular vein cannulas were simultaneously opened to allow complete washout of the blood. Pneumothorax was created by opening slits through the diaphragm. Because the temperature and pressure of the system stabilized within 20 min, all of the experiments were performed 30 min after starting perfusion at a constant rate of 40 ml/min. The criteria for liver viability included gross appearance and stability of perfusion pressure.

### 2.5 Color microsphere method for portosystemic shunting degree analysis

The degree of portosystemic shunting was determined using the technique described by Chojkier and Groszmann and substituting color for radioactive microspheres; 30,000 15‐μm yellow microspheres (Dye Track; Triton Technology) were slowly injected into the spleen.^24^ The rats were euthanized, and the livers and lungs were dissected. The number of microspheres in each tissue was then determined: 3000 blue microspheres (Dye Track; Triton Technology) were added to each tube as an internal control. Tissue was digested overnight with 1 M KOH at 60°C and thoroughly sonicated. After centrifugation, the supernatant was removed, and the pellets were washed once with 10% Triton X‐100 and twice with acidified ethanol. At the end of the process, a minimum pellet containing the microspheres was allowed to dry overnight. The color of the microspheres was diluted with 200 μl of acidified Cellosolve acetate (Spectrum Chemicals). The absorbance of the solution was read at 448‐nm wavelength (yellow) and 670‐nm wavelength (blue) in a spectrophotometer (Shimadzu, ), and the number of microspheres was calculated by comparison with standards. Spillover between wavelengths was corrected using the matrix inversion technique. Portosystemic shunting was calculated as the number of lung microspheres/(liver microspheres and lung microspheres). Assuming a worst‐case scenario in which two‐thirds of the microspheres remained trapped in the spleen, this technique could detect a minimum shunt of 3.5%. Studies using color microspheres have been shown to provide results similar to those using radioactive microspheres.^25^

### 2.6 Immunofluorescent study for mesenteric vascular density

Mesenteric angiogenesis was quantified using CD31‐labelled microvascular networks in rat mesenteric connective tissue windows according to a previous study.^26^ At least four mesenteric windows (wedge‐shaped regions of connective tissue bordered by the intestinal wall and ileal blood vessel pairs) were dissected free in each rat, washed in PBS, dried on gelatin slides and fixed in 100% MeOH (−20℃ for 30 min). The slides were incubated overnight at 4℃ with the primary antibody mouse anti‐rat CD31‐biotin [1:200; AbD Serotec, Oxford, UK]. Secondary antibodies [CY2‐conjugated streptavidin, 1:1000; Jackson ImmunoResearch, West Grove, PA, USA] were then applied for 1 h at room temperature. At least four sets of data were obtained for each mesenteric window. (100×)‐magnification immunofluorescent images were assessed using an upright fluorescent microscope (AX80, Olympus, Japan) with a charge‐couple device (QICAM, High‐performance IEEE 1394 FireWireTM Digital CCD Camera, Q IMAGING, BD, Canada) and thresholded using Image J software (available for download from the National Institutes of Health (NIH, http://rsb.info.nih.gov/ij/). The vascular area was measured automatically using the histogram function.

### 2.7 Hepatic Sirius red and haematoxylin and eosin (H&E) staining

Livers were fixed in 10% formalin, embedded in paraffin, sectioned at 5 μm, and stained with H&E and Sirius red, respectively, using a staining kit (Polysciences Inc.). Image J software was used to measure the percentage of Sirius red‐stained area. Briefly, a grayscale image was used, and the Sirius red‐stained collagen was isolated using the thresholding function. The thresholded area was then measured and presented as the percentage of thresholded area per image.

### 2.8 Western blotting analysis

Tissue samples were immediately frozen in liquid nitrogen and stored at −80℃ until required. Protein extracts were obtained by pulverization in a grinder with liquid nitrogen, followed by the addition of 1 ml of lysis buffer (PBS containing 1% Nonidet P‐40, 0.5% sodium deoxycholate, 0.1% sodium dodecyl sulfate (SDS), and 0.05% protease inhibitor cocktail solution (Roche Diagnostics GmbH)) for each 100 mg of powdered sample. The protein concentration was determined for each sample. An aliquot of 20–40 µg protein from each sample dissolved in sample buffer (63 mmol/L of Tris‐HCL, pH 6.8, containing 2% SDS, 10% glycerol, 5% 2‐mercaptoethanol and 0.005% bromophenol blue) and 10 µg positive control were separated on denaturing SDS‐10% polyacrylamide gels by electrophoresis (Mini‐PROTEAN® 3 Cell, Bio‐Rad Laboratories). Prestained protein markers (SDS‐PAGE Standards, Bio‐Rad Laboratories) were used for molecular weight determination. Proteins were then transferred to a polyvinylidene difluoride membrane (Immum‐BlotTM PVDF Membrane, Bio‐Rad Laboratories) using a semi‐dry electroblotting system (Trans‐Blot® SD Semi‐dry Electrophoretic Transfer Cell, Bio‐Rad Laboratories) for 1.5 h at 4℃. To block non‐specific binding, the membranes were blocked for 30 min with 3% non‐fat dry milk in TBS‐T, pH 7.4 (25 mmol/l Tris base‐137 mmol/l NaCl‐2.7 mmol/l KCL‐1% Tween 20). Blots were incubated with the primary antibody, diluted with 3% non‐fat dry milk in TBS‐T for 90 min at room temperature and washed. The blots were then incubated for 90 min with the secondary antibody and washed. l. The specific proteins were detected by enhanced chemiluminescence (Immobilon Western Chemiluminescent horseradish peroxidase Substrate; Merk Millipore Co.). Using a computer‐assisted video densitometer and digitalized system (BioSpectrum 600 Imaging System; Ultra‐Violet Products Ltd.), the blots were scanned and photographed, then the signal intensity (integral volume) of the appropriate band was determined.

### 2.9 Drugs

Minocycline was purchased from Yung Shin Pharmaceutical Company, Taiwan. SB‐3CT was purchased from Merck KGaA (Darmstadt, Germany). All solutions were freshly prepared on the days of the experiments.

### 2.10 Statistical analysis

All results are expressed as mean ± standard error of the mean. Changes in perfusion pressure (mmHg) compared to baseline were calculated for each concentration in each preparation. The Shapiro‐Wilk normality test showed that almost all of the data were normally distributed. Statistical analyses were performed using an unpaired Student's *t*‐test or one‐way ANOVA with LSD post‐hoc test as appropriate. Results were considered to be statistically significant at a two‐tailed P‐value of less than 0.05.

## 3 RESULTS

### 3.1 Minocycline: Non‐selective MMP inhibitor

#### 3.1.1 BDL induced liver cirrhosis, portal hypertension and hyperdynamic circulation

Compared with the sham operation, BDL successfully induced liver cirrhosis with portal hypertension in the rats (Table 1, PP, Sham‐V (vehicle) vs. BDL‐V, *p* < 0.001). In addition, the BDL rats had features of hyperdynamic circulation, including lower MAP (*p* = 0.005), higher CI (*p* < 0.001), lower SVR (*p* < 0.001), higher SMA flow (*p* < 0.001) and lower SMA resistance (*p* < 0.001). Plasma biochemistry parameters revealed cholestasis and significant liver injury in the BDL rats (ALT, AST, total bilirubin, *p* < 0.001). However, renal function was not affected in the BDL rats (BUN, creatinine, *p* > 0.05).

**TABLE 1:** Hemodynamic and plasma biochemistry parameters in the sham and BDL (bile duct ligation) rats receiving vehicle or the nonselective MMP inhibitor minocycline

	Sham‐V	Sham‐M	BDL‐V	BDL‐M
	*n* = 6	*n* = 7	*n* = 10	*n* = 8
BW (g)	390 ± 10	424 ± 8	377 ± 12	383 ± 13
PP (mmHg)	9.1 ± 0.2	9.1 ± 0.2	17.2 ± 0.5^§^	15.3 ± 0.6^†^
Systemic circulation				
MAP (mmHg)	132 ± 6	119 ± 2	114 ± 5^§^	108 ± 3
HR (beats/min)	381 ± 21	331 ± 12	350 ± 17	345 ± 16
CI (ml/min/100 g)	27.7 ± 1.0	25.3 ± 1.7	37.1 ± 1.5^§^	30.5 ± 1.2^†^
SVR (mmHg/ml/min/100 g)	4.8 ± 0.2	4.8 ± 0.2	3.1 ± 0.2^§^	3.6 ± 0.2
Splanchnic system				
SMA flow (ml/min/100 g)	5.2 ± 0.3	4.7 ± 0.2	8.6 ± 0.4^§^	7.5 ± 0.3*
SMA resistance (mmHg/ml^/^min/100 g)	24.1 ± 2.0	23.8 ± 0.9	11.4 ± 0.4^§^	12.5 ± 0.6
Plasma biochemistry				
ALT (U/L)	47 ± 3	42 ± 2	188 ± 22^§^	161 ± 28
AST (U/L)	89 ± 6	88 ± 6	857 ± 70^§^	760 ± 108
Total bilirubin (mg/dl)	<0.15	<0.15	8.5 ± 0.4^§^	7.8 ± 0.2
BUN (mg/dl)	23 ± 2	24 ± 3	23 ± 2	26 ± 1
Creatinine (mg/dl)	0.43 ± 0.04	0.49 ± 0.09	0.46 ± 0.03	0.49 ± 0.02

Abbreviations: ALT, alanine transaminase; BDL, bile duct ligation; BUN, blood urea nitrogen; BW, body weight; CI, cardiac index; HR, heart rate; M, minocycline; MAP, mean arterial pressure; PP, portal pressure; SMA, superior mesenteric artery; SVR, systemic vascular resistance; V, vehicle.

*p < 0.05, ^†^ *p* < 0.01, vehicle groups compared to corresponding minocycline groups.

^§^ *p* < 0.05, sham groups compared to corresponding BDL groups.

#### 3.1.2 Body weight and hemodynamic parameters post treatment

Minocycline significantly reduced PP in the BDL rats (BDL‐V vs. BDL‐M (minocycline), *p* = 0.008), and also significantly decreased SMA flow and CI (SMA flow: *p* = 0.023; CI: *p* = 0.002) (Table 1). However, minocycline did not affect BW, MAP, heart rate, SVR or SMA resistance in the BDL rats. In addition, minocycline did not influence BW or systemic hemodynamic parameters compared with vehicle in the sham‐operated rats. Furthermore, minocycline did not influence biochemistry parameters in either the sham or BDL rats.

#### 3.1.3 Severity of shunting and mesenteric angiogenesis

**FIGURE 1:**
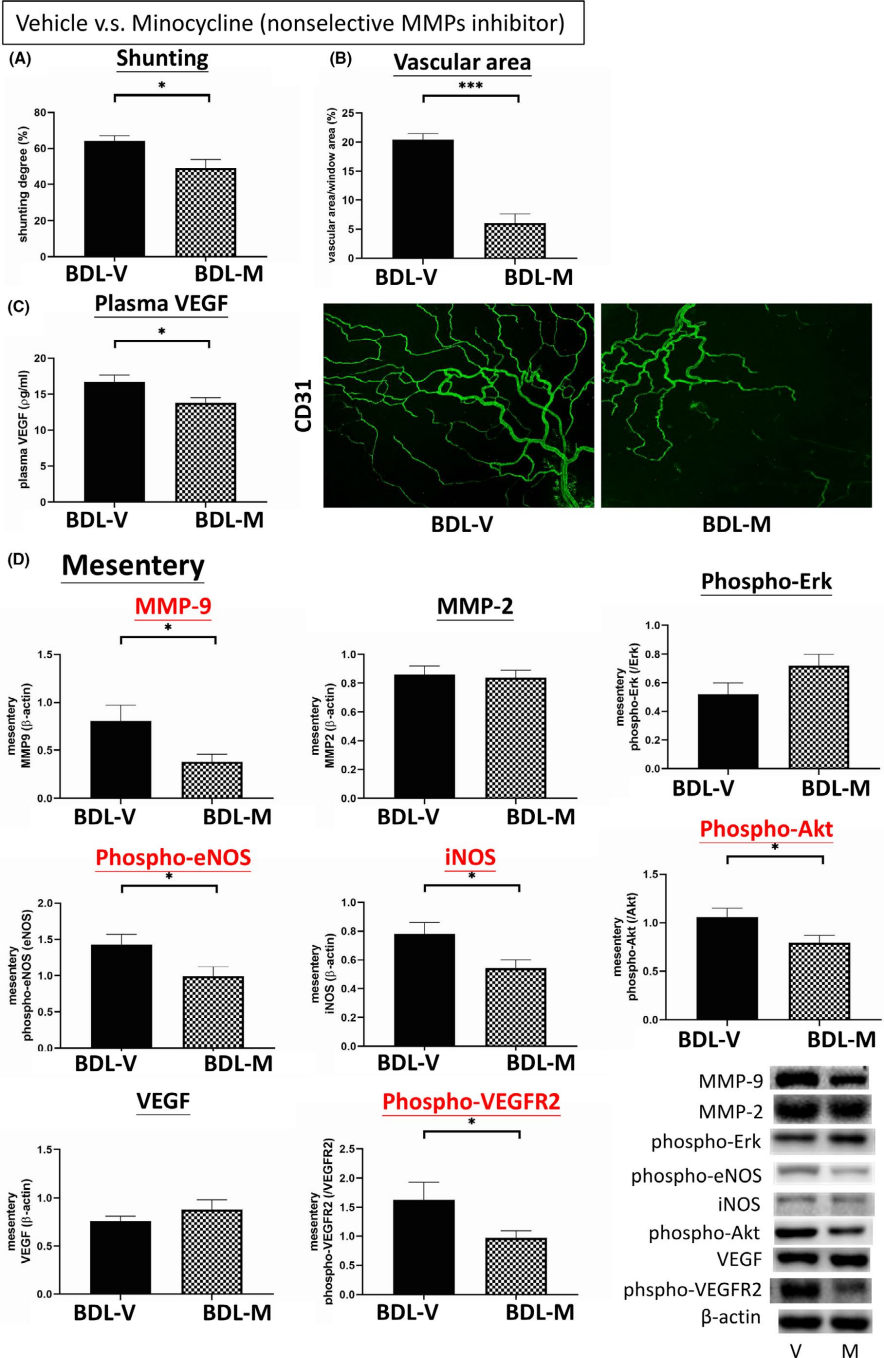
Effects of the non‐selective matrix metalloproteinase (MMP) inhibitor minocycline on extrahepatic systems. (A) Minocycline effectively reduced portosystemic shunting in cirrhotic rats. (B) The mesenteric vascular densities, as represented by vascular area, decreased significantly in the minocycline‐treated group. This suggested that minocycline suppressed mesenteric angiogenesis. (C) The plasma expression of VEGF was decreased in the minocycline‐treated BDL‐cirrhotic group. (D) Minocycline significantly downregulated mesenteric MMP‐9, phospho‐VEGFR2, phospho‐eNOS, iNOS and phospho‐Akt expressions in BDL‐cirrhotic rats. These results suggested that minocycline attenuated mesenteric angiogenesis through downregulation of the VEGF pathway. BDL‐V: BDL‐vehicle; BDL‐M: BDL‐minocycline. *p < 0.05; **p < 0.01; ***p < 0.001

Figure 1A shows the severity of portosystemic shunting in the BDL rats treated with vehicle or minocycline. Minocycline significantly reduced the degree of shunting in the BDL rats compared with vehicle (BDL‐V vs. BDL‐M (%): 64.10 ± 2.98 vs. 49.05 ± 4.80, *p* = 0.013, *n* = 11, 9). Figure 1B shows that minocycline decreased mesenteric vascular density in the BDL rats compared with vehicle (vascular area/ mesenteric area (%): 20.4 ± 1.04 vs. 6.05 ± 1.60, *p* < 0.001, *n* = 6, 6). The representative CD31 immunofluorescence images of mesenteric windows are shown below.

Figure 1C shows the plasma VEGF concentration in the BDL rats treated with vehicle or minocycline. Compared with vehicle, minocycline significantly decreased the plasma VEGF concentration (BDL‐V vs. BDL‐M (ρg/ml): 16.71 ± 0.96 vs. 13.81 ± 0.73, *p* = 0.028, *n* = 9, 9). Figure 1D shows the mesenteric protein expressions of the BDL groups (*n* = 11, 11). Minocycline significantly downregulated the mesenteric protein expressions of MMP‐9 (0.8043 ± 0.1676 vs. 0.3838 ± 0.0761, *p* = 0.033), iNOS (0.7789 ± 0.0806 vs. 0.5378 ± 0.0616, *p* = 0.030), phospho‐eNOS (1.4287 ± 0.1357 vs. 0.9857 ± 0.1347, *p* = 0.034), phospho‐Akt (1.0565 ± 0.0878 vs. 0.7928 ± 0.0776, *p* = 0.044) and phospho‐VEGFR2 (1.6277 ± 0.2960 vs. 0.9717 ± 0.1181, *p* = 0.048). The expressions of MMP‐2, VEGF and phospho‐Erk were not significantly different between groups. The representative blots are shown below.

#### 3.1.4 Splanchnic responsiveness to vasoconstrictors

Figure S1A shows the changes in SMA territory perfusion pressure to arginine vasopressin (AVP) in the sham and BDL rats treated with vehicle or minocycline. Compared to the sham rats, the BDL rats had lower perfusion pressure changes with vasoconstrictors (sham‐V vs. BDL‐V, *p* = 0.035, *n* = 5, 6), suggesting a poorer splanchnic vasoconstrictive response in the cirrhotic rats. Minocycline did not influence splanchnic vascular contractility in the cirrhotic rats (BDL‐V vs. BDL‐M, *p* = 0.726, *n* = 6, 5).

Figure S1B shows the SMA protein expressions of the BDL‐V and BDL‐M groups (*n* = 8, 10). Minocycline significantly downregulated the SMA protein expressions of MMP‐9 (1.2540 ± 0.2131 vs. 0.7292 ± 0.0986, *p* = 0.035), MMP‐2 (0.8656 ± 0.1396 vs. 0.3986 ± 0.1142, *p* = 0.021) phospho‐eNOS (1.9154 ± 0.4405 vs. 0.7714 ± 0.1599, *p* = 0.042), COX‐1 (1.4480 ± 0.1638 vs. 0.9562 ± 0.1390, *p* = 0.035), and COX‐2 (1.3710 ± 0.2052 vs. 0.7425 ± 0.1232, *p* = 0.014).

#### 3.1.5 Hepatic vascular responsiveness to vasoconstrictors and fibrosis

**FIGURE 2:**
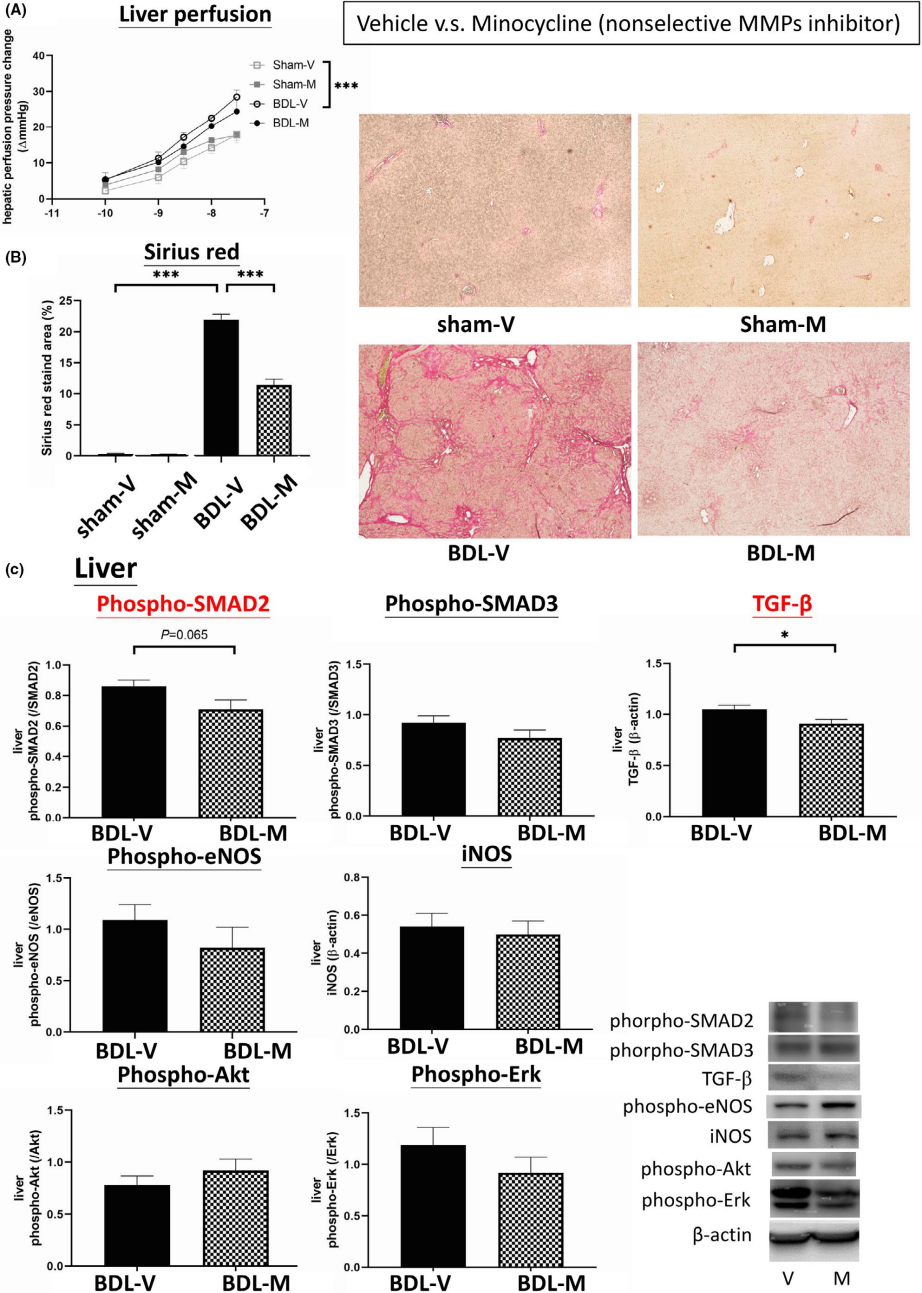
Effects of the non‐selective MMP inhibitor minocycline on the hepatic system. (A) BDL rats had significantly higher perfusion pressure changes with ET‐1 compared to the sham group. These results indicated enhanced vasoconstriction in the hepatic vascular system in the cirrhotic rats. Minocycline did not affect the hepatic vasoresponsiveness in cirrhotic rats. (B) Liver fibrosis determined by Sirius red‐stained area. The liver fibrotic area was significantly increased in the BDL‐V group, and the effect was reversed by minocycline treatment (BDL‐M). (C) The protein expressions of liver fibrogenesis factors in the BDL rats treated with vehicle or minocycline. Minocycline downregulated the protein expressions of hepatic TGF‐β and phospho‐SMAD2. sham‐V: sham‐vehicle; sham‐M: sham‐minocycline; *p < 0.05; **p < 0.01; ***p < 0.001

Figure 2A reveals the hepatic vascular responsiveness to endothelin‐1 (ET‐1) in the sham and BDL rats treated with vehicle or minocycline. The BDL rats had significantly higher perfusion pressure changes with ET‐1 compared to the sham rats (sham‐V vs. BDL‐V, *p* < 0.001, *n* = 7, 10), indicating enhanced vasoconstriction in the hepatic vascular system of the cirrhotic rats. Minocycline did not affect hepatic vascular responsiveness in the cirrhotic rats (BDL‐V vs. BDL‐M, *p* = 0.258, *n* = 10, 7).

Figure 2B reveals the Sirius red‐stained fibrotic area of the livers: (sham‐V vs. sham‐M vs. BDL‐V vs. BDL‐M, %): 0.32 ± 0.09 vs. 0.28 ± 0.03 vs. 21.96 ± 0.88 vs. 11.41 ± 0.93, sham‐V vs. BDL‐V: *p* < 0.001; BDL‐V vs. BDL‐M: *p* < 0.001). Minocycline significantly decreased the liver fibrotic area in the BDL rats but not in the sham rats.

Figure 2C shows the hepatic protein expressions in the BDL rats treated with vehicle or minocycline (*n* = 7, 5). Minocycline significantly downregulated the protein expression of TGF‐β (1.0460 ± 0.0382 vs. 0.9060 ± 0.0372, *p* = 0.030). There was a trend toward a decreased expression of phospho‐SMAD2 (0.8583 ± 0.0383 vs. 0.7145 ± 0.0598, *p* = 0.065). However, there were no significant differences in the expressions of phospho‐eNOS, iNOS, phospho‐Akt, phospho‐Erk or phospho‐SMAD3 between groups (*p* > 0.05). The representative blots are shown below.

### 3.2 SB‐3CT: MMP‐2 and MMP‐9 inhibitor

#### 3.2.1 Body weight and hemodynamic parameters post treatment

The BW and hemodynamic parameters in the sham and BDL rats treated with vehicle or SB‐3CT are shown in Table 2. In the BDL rats, SB‐3CT did not affect BW, MAP or SVR, but reduced CI (*p* < 0.001).

**TABLE 2:** Hemodynamic and plasma biochemistry parameters in the sham and BDL (bile duct ligation) rats receiving vehicle or the MMP‐2/MMP‐9 selective inhibitor SB‐3CT

	Sham‐V	Sham‐S	BDL‐V	BDL‐S
	*n* = 6	*n* = 7	*n* = 8	*n* = 8
BW (g)	406 ± 11	380 ± 16	368 ± 9^§^	374 ± 9
PP (mmHg)	9.0 ± 0.5	8.5 ± 0.4	16.7 ± 0.9^§^	13.9 ± 1.1*
Systemic circulation				
MAP (mmHg)	130 ± 7	132 ± 9	112 ± 3	110 ± 4
HR (beats/min)	359 ± 26	399 ± 27	334 ± 12	374 ± 9
CI (ml/min/100 g)	25.6 ± 1.1	26.1 ± 1.7	40.7 ± 1.8^§^	32.6 ± 0.9^‡^
SVR (mmHg/ml/min/100 g)	5.1 ± 0.3	5.2 ± 0.5	2.8 ± 0.2^§^	3.4 ± 0.2
Splanchnic system				
SMA flow (ml/min/100 g)	5.9 ± 0.5	5.5 ± 0.5	8.9 ± 0.5^§^	7.0 ± 0.2^†^
SMA resistance (mmHg/ml^/^min/100 g)	21.2 ± 1.8	22.9 ± 1.9	11.2 ± 1.1^§^	13.8 ± 0.7
Plasma biochemistry				
ALT (U/L)	48 ± 2	44 ± 3	107 ± 13^§^	155 ± 36
AST (U/L)	78 ± 3	88 ± 6	635 ± 89^§^	595 ± 85
Total bilirubin (mg/dl)	<0.15	<0.15	7.4 ± 1.4^§^	8.0 ± 0.3
BUN (mg/dl)	19 ± 1	19 ± 1	23 ± 1	21 ± 3
Creatinine (mg/dl)	0.34 ± 0.04	0.37 ± 0.01	0.35 ± 0.02	0.39 ± 0.03

Abbreviations: ALT, alanine transaminase; BDL, bile duct ligation; BUN, blood urea nitrogen; BW, body weight; CI, cardiac index; HR, heart rate; MAP, mean arterial pressure; PP, portal pressure; S, SB‐3CT; SMA, superior mesenteric artery; SVR, systemic vascular resistance; V, vehicle.

*p < 0.05, ^†^ *p* < 0.01, ^‡^ *p* < 0.001, vehicle groups compared to minocycline groups.

^§^ *p* < 0.05, sham groups compared to BDL groups.

Regarding the portal haemodynamics, SB‐3CT reduced PP (*p* = 0.019) and SMA flow (*p* = 0.003) without affecting SMA resistance compared with vehicle. SB‐3CT did not affect the BW or haemodynamic parameters compared with vehicle in the sham rats. In addition, SB‐3CT did not influence the biochemistry parameters compared with vehicle in the sham and BDL rats.

#### 3.2.2 Severity of shunting, mesenteric vascular density and plasma VEGF concentration

**FIGURE 3:**
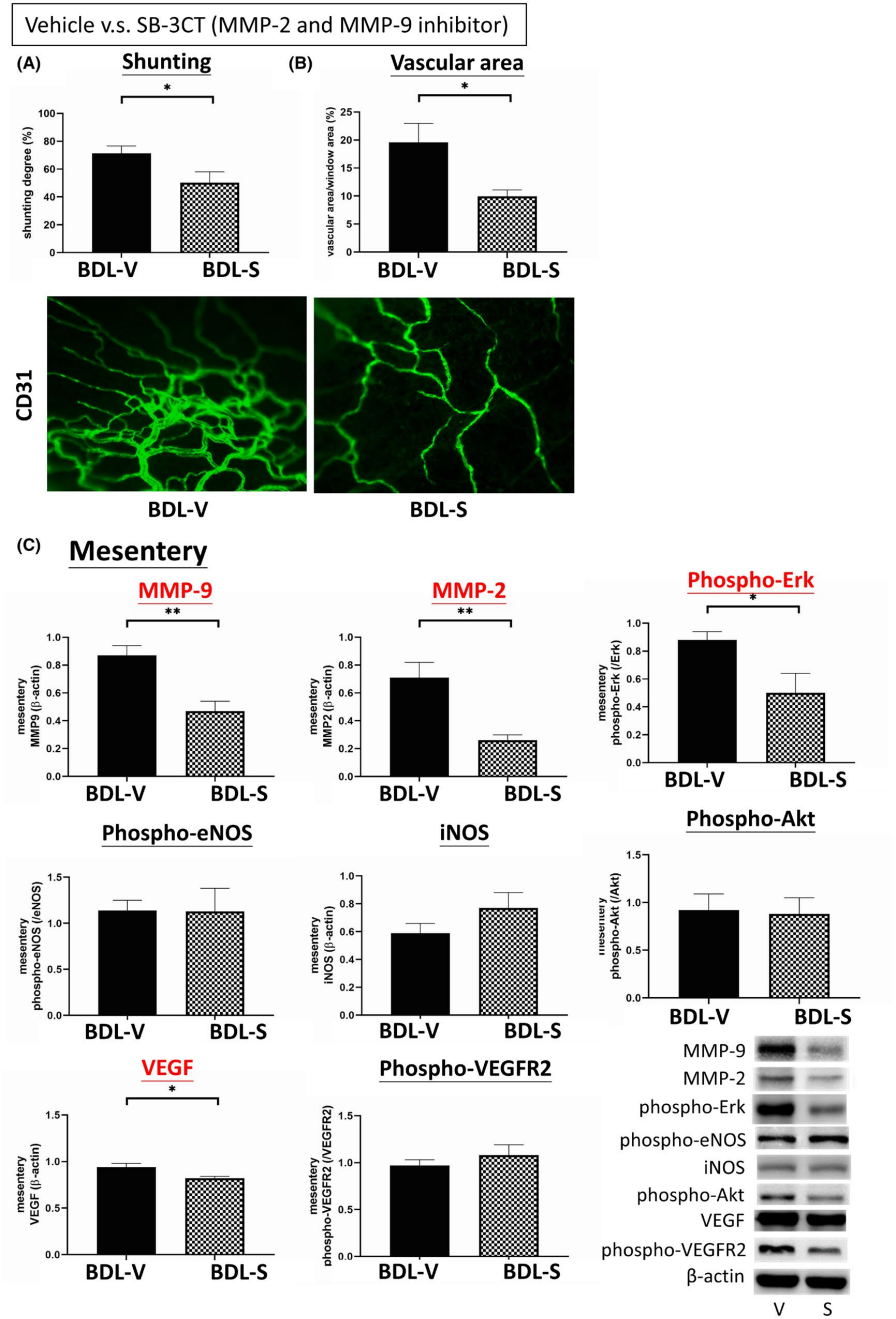
Effects of the MMP‐2/MMP‐9 inhibitor SB‐3CT on extrahepatic systems. (A) SB‐3CT reduced the degree of shunting compared with vehicle in the BDL rats. (B) Mesenteric vascular density was decreased in the SB‐3CT‐treated group. (C) The protein expressions of mesenteric angiogenesis factors in the BDL rats treated with vehicle or SB‐3CT. SB‐3CT significantly downregulated the protein expressions of mesenteric MMP‐9, MMP‐2, VEGF and phospho‐Erk. BDL‐V: BDL‐vehicle; BDL‐S: BDL‐SB‐3CT. *p < 0.05; **p < 0.01; ***p < 0.001

Figure 3A shows the severity of shunting in the BDL rats treated with vehicle or SB‐3CT. SB‐3CT significantly reduced the degree of shunting in the BDL rats compared with vehicle (BDL‐V vs. BDL‐S (SB‐3CT) (%): 71.27 ± 5.31 vs. 50.18 ± 7.83, *p* = 0.043, *n* = 7, 5). Figure 3B reveals that SB‐3CT reduced mesenteric vascular density in the BDL rats compared with vehicle (vascular area/mesenteric area (%): 19.58 ± 3.39 vs. 9.93 ± 1.17, *p* = 0.044, *n* = 5, 5).

Figure 3C reveals the mesenteric protein expressions in the vehicle‐ and SB‐3CT‐treated BDL rats (*n* = 6, 6). SB‐3CT significantly downregulated the protein expressions of VEGF (0.9357 ± 0.0432 vs. 0.8203 ± 0.0194, *p* = 0.029), phospho‐Erk (0.8813 ± 0.0648 vs. 0.4998 ± 0.1351, *p* = 0.034), MMP‐2 (0.7147 ± 0.1085 vs. 0.2616 ± 0.0364, *p* = 0.007), and MMP‐9 (0.8747 ± 0.0682 vs. 0.4690 ± 0.0654, *p* = 0.003). However, there were no significant differences in phospho‐eNOS, iNOS, phosphor‐Akt and phosphor‐VEGFR2 expressions.

#### 3.2.3 SMA territory and hepatic vascular responsiveness to vasoconstrictors

Figure S2A shows the changes in SMA vascular bed perfusion pressure with AVP in the BDL rats treated with vehicle or SB‐3CT. In both sham and BDL rats, the perfusion pressure changes with AVP were not significantly different between the vehicle‐ and SB‐3CT‐treated groups. Figure S2B shows the hepatic vascular responsiveness to ET‐1 in the sham and BDL rats treated with vehicle or SB‐3CT. The BDL rats had a significantly higher response to ET‐1 compared to the sham rats (Sham‐V vs. BDL‐V, *p* = 0.006, *n* = 5, 6). SB‐3CT did not affect hepatic vascular responsiveness in the cirrhotic rats (BDL‐V vs. BDL‐M, *p* = 0.176, *n* = 6, 5).

#### 3.2.4 Liver fibrosis

**FIGURE 4:**
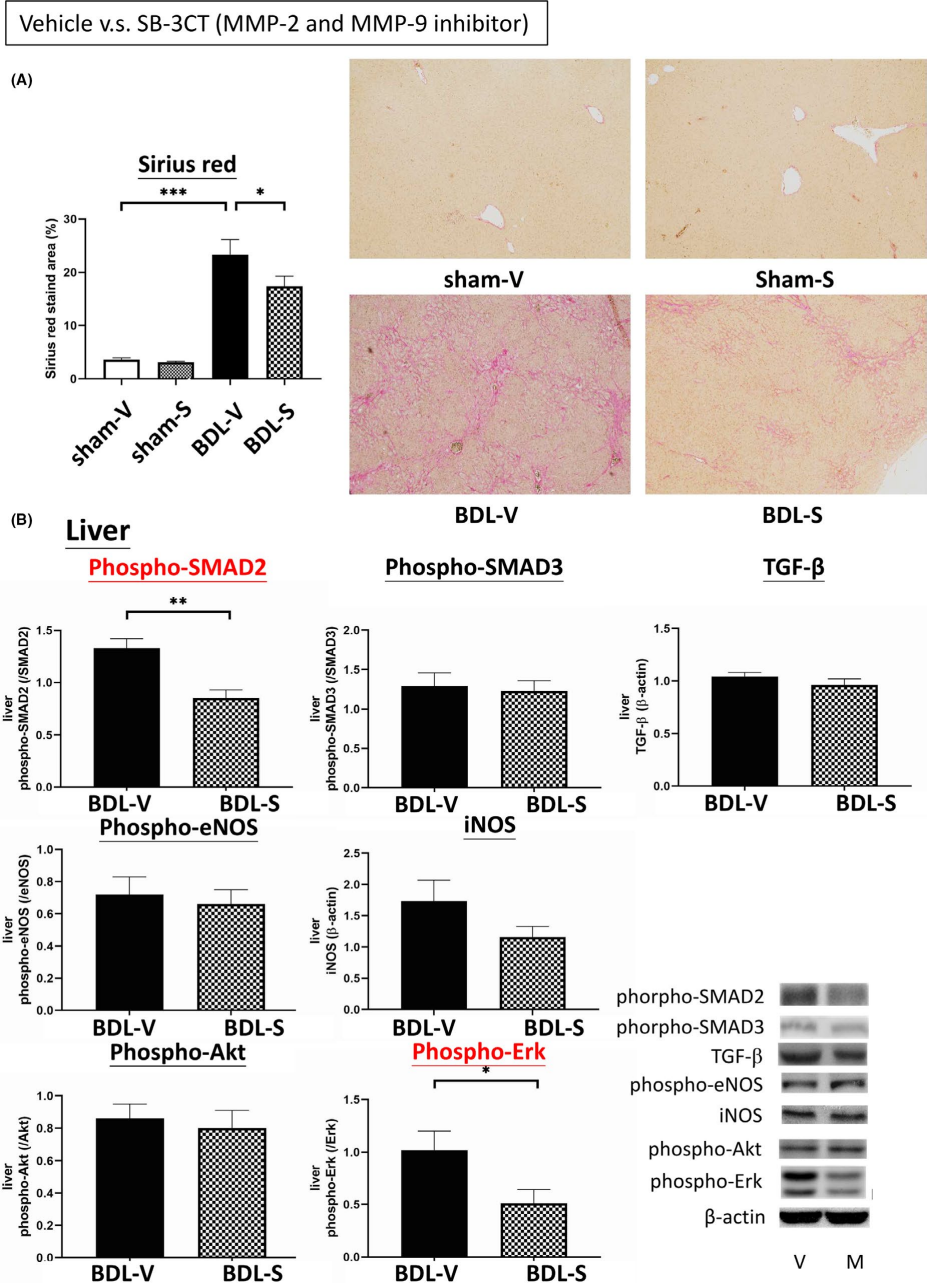
Effects of the MMP‐2/MMP‐9 inhibitor SB‐3CT on liver fibrosis. (A) SB‐3CT significantly reduced the Sirius‐red‐stained area in the BDL rats. (B) The protein expressions of liver fibrogenesis factors in the BDL rats treated with vehicle or SB‐3CT. SB‐3CT significantly downregulated the expressions of phospho‐SMAD2 and phospho‐Erk in the BDL rats. *p < 0.05; **p < 0.01; ***p < 0.001

Figure 4A reveals the Sirius red‐stained fibrotic area of the livers in the vehicle‐ and SB‐3CT‐treated BDL rats. Compared with vehicle, SB‐3CT significantly reduced the Sirius red‐stained area in the BDL groups (BDL‐V vs. BDL‐S: 23.31 ± 2.82 vs. 17.38 ± 1.92, *p* = 0.028). The representative images of Sirius red staining are shown below, showing that the livers of the BDL rats had prominent liver fibrosis, and that this was reversed by SB‐3CT treatment.

Figure 4B shows the hepatic fibrogenesis‐related protein expressions in vehicle‐ and SB‐3CT‐treated BDL rats (*n* = 6, 6). SB‐3CT significantly downregulated the expressions of phospho‐SMAD2 (1.3308 ± 0.0881 vs. 0.8536 ± 0.0817, *p* = 0.003) and phospho‐Erk (0.6007 ± 0.0392 vs. 0.3424 ± 0.0353, *p* = 0.003).

### 3.3 MMP‐9 knockout mice

#### 3.3.1 Body weight and haemodynamics

**FIGURE 5:**
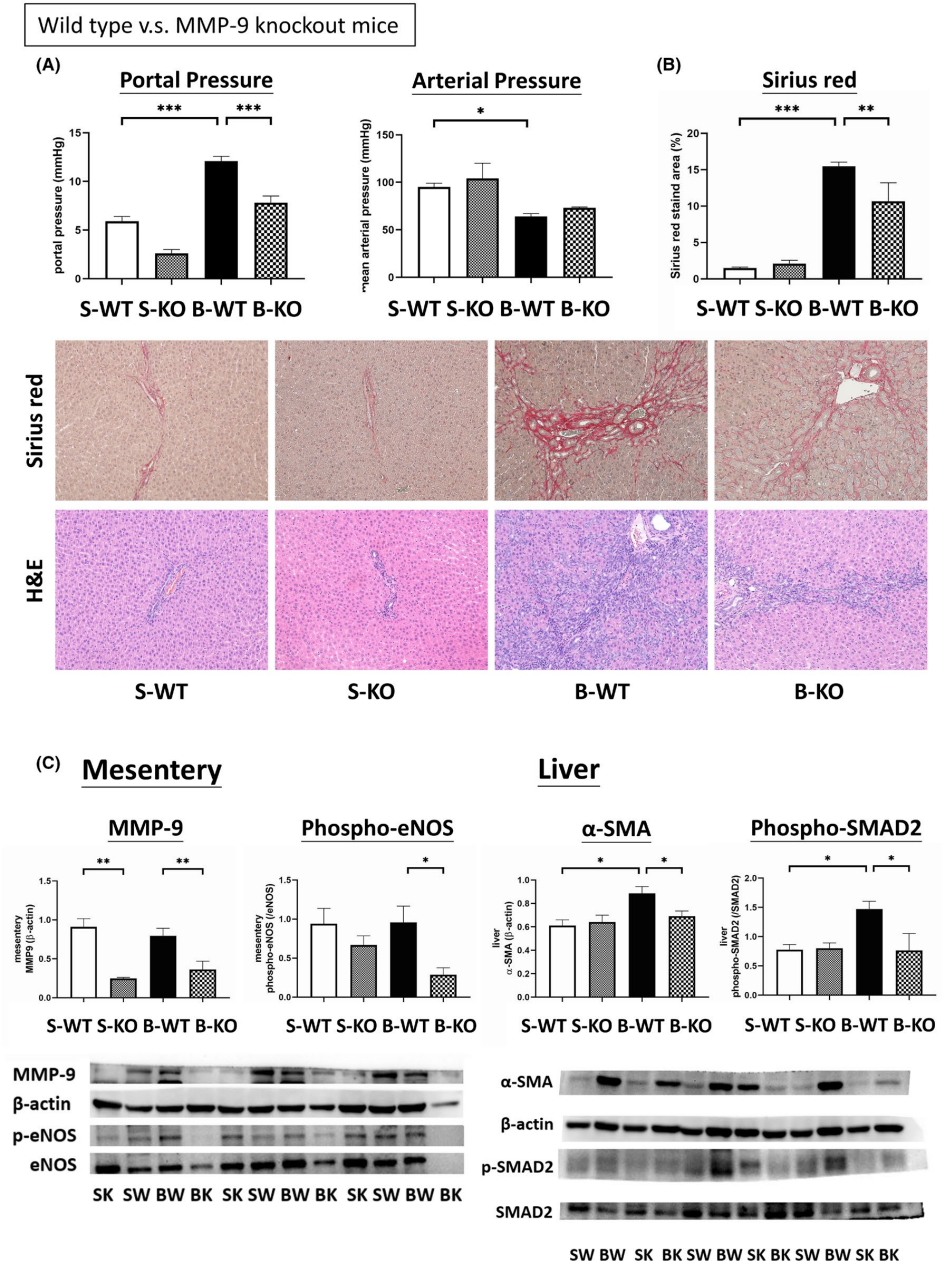
Effects of MMP‐9 knockout (KO) on liver cirrhosis and portal hypertension. (A) Portal pressure and arterial pressure in wild‐type (WT) or MMP‐9 KO mice receiving sham (S) or BDL (B) operations. BDL induced portal hypertension in the mice. The MMP‐9 KO mice had significantly lower portal pressure compared to the WT mice that received BDL operations. (B) BDL significantly increased the Sirius red‐stained fibrotic area in both the WT and MMP‐9 KO mice. The B‐KO mice had a significantly lower Sirius red‐stained area than the B‐WT mice. Middle and lower panels are representative Sirius red and H&E staining images of livers. (C) MMP‐9 KO mice had significantly lower MMP‐9 expressions in both the sham and BDL groups compared to the corresponding WT groups. MMP‐9 KO‐BDL mice had a significantly lower mesenteric phospho‐eNOS expression than the WT‐BDL mice. BDL upregulated the protein expressions of hepatic α‐SMA and phospho‐SMAD2 in WT mice but not in MMP‐9 KO mice. S‐WT: Sham‐WT; S‐KO: Sham‐KO; B‐WT: BDL‐WT; B‐KO: BDL‐KO. *p < 0.05, **p < 0.005, ***p < 0. 001

Figure 5A shows the hemodynamic changes in the WT or MMP‐9 knockout (KO) mice that received sham or BDL operations. BDL reduced MAP and elevated PP (*p* < 0.05) in the WT mice, indicating that BDL induced the systemic and splanchnic hemodynamic features of portal hypertension. MMP‐9 KO BDL mice had a significantly lower PP compared with WT BDL mice ((mmHg): 12.1 ± 0.5 vs. 7.8 ± 0.7, *p* = 0.007, *n* = 6, 5).

#### 3.3.2 Serum liver and kidney biochemistry parameters

The serum liver and kidney biochemistry parameters in all groups are shown in Table S1. BDL significantly increased ALT, AST and total bilirubin levels in both the WT and MMP‐9 KO mice. BUN and creatinine levels were not significantly different among the four groups.

#### 3.3.3 Histology

Figure 5B depicts representative Sirius red and H&E staining images of the livers in the WT and MMP‐9‐KO mice that received BDL or sham operations. BDL significantly increased the Sirius red‐stained fibrotic area in the WT and MMP‐9 KO mice compared with their corresponding sham groups. The KO mice had a significantly lower Sirius red‐stained area compared with the WT mice (%: sham‐WT (S‐WT) vs. sham‐KO (S‐KO) vs. BDL‐WT (B‐WT) vs. BDL‐KO (B‐KO): 1.50 ± 0.13 vs. 2.11 ± 0.45 vs. 15.48 ± 0.56 vs. 10.65 ± 2.55, sham‐WT vs. BDL‐WT: *p* < 0.001, BDL‐WT vs. BDL‐KO: *p* = 0.004).

#### 3.3.4 Western blot

Figure 5C shows the mesenteric and liver protein expressions. The MMP‐9 protein expression was significantly lower in the KO mice (/β‐actin: sham‐WT vs. sham‐KO vs. BDL‐WT vs. BDL‐KO: 0.9097 ± 0.1060 vs. 0.2483 ± 0.0133 vs. 0.7950 ± 0.0968 vs. 0.3630 ± 0.1056, Sham‐WT vs. Sham‐KO: *p* = 0.001, BDL‐WT vs. BDL‐KO: *p* = 0.009). The MMP‐9 KO‐BDL mice had a significantly lower mesenteric phospho‐eNOS expression than the WT‐BDL and MMP‐9 KO‐sham mice (/eNOS: 0.9410 ± 0.1960 vs. 0.6678 ± 0.1175 vs. 0.9584 ± 0.2089 vs. 0.2875 ± 0.0894, BDL‐WT vs. BDL‐KO: *p* = 0.039).

BDL upregulated hepatic α‐SMA protein expression in the livers of the WT mice. The BDL‐KO mice had a significantly lower α‐SMA protein expression than the BDL‐WT mice (/β‐actin: 0.6113 ± 0.0495 vs. 0.6410 ± 0.0582 vs. 0.8863 ± 0.0571 vs. 0.6243 ± 0.0992, sham‐WT vs. BDL‐WT: *p* = 0.022, BDL‐WT vs. BDL‐KO: *p* = 0.027). Consistent with the α‐SMA results, phospho‐SMAD2 was upregulated in the BDL‐WT mice and downregulated in the MMP‐9 KO mice (/β‐actin: 0.7762 ± 0.0877 vs. 0.7999 ± 0.0908 vs. 1.4702 ± 0.1333 vs. 0.7623 ± 0.2909, sham‐WT vs. BDL‐WT: *p* = 0.021, BDL‐WT vs. BDL‐KO: *p* = 0.020).

## 4 DISCUSSION

In this study, all three series of experiments consistently demonstrated that MMP‐9 suppression alleviated portal hypertension. Minocycline, a nonselective MMP inhibitor, and 3B‐SCT, an MMP‐2 and MMP‐9 inhibitor, significantly attenuated portal hypertension in rats with BDL‐induced liver cirrhosis. Furthermore, MMP‐9 gene deletion effectively ameliorated portal hypertension in BDL mice. These results suggest that MMP‐9 inhibition is a reasonable method to control portal hypertension.

Minocycline, a nonselective MMP inhibitor, attenuated portal hypertension both through extrahepatic and intrahepatic mechanisms. Portal pressure is determined by three main factors: portal blood inflow as assessed by SMA flow, hepatic resistance, and portosystemic collateral vascular resistance.^2^, ^3^ Since in a vascular system pressure is positively correlated with flow, the minocycline‐induced reduction in SMA flow played at least a partial role in reducing PP.

Splanchnic hyperdynamic circulation is triggered by overt vasodilatation and abnormal angiogenesis.^3^ Of note, SMA resistance and systemic vascular resistance were not influenced by minocycline in the current study. In addition, minocycline did not modify vascular responsiveness to AVP in SMA territory vascular beds. Furthermore, the hepatic vascular responsiveness to ET‐1 was not affected in either the sham or BDL rats. The beneficial effects of minocycline on PP, therefore, seem to be related to structural changes rather than functional changes. Consistent with this hypothesis, we found that minocycline significantly decreased the mesenteric vascular area in the BDL rats. This could be related to the downregulation of pro‐angiogenic factors, since minocycline significantly downregulated the expressions of mesenteric phospho‐VEGFR2, phospho‐eNOS, iNOS and phospho‐Akt and reduced plasma levels of VEGF. Taken together, the nonselective MMP inhibitor decreased portal pressure mainly through ameliorating extrahepatic angiogenesis but not vascular resistance.

Interestingly, the more specific MMP‐2/MMP‐9 inhibitor SB‐3CT also decreased PP and SMA flow in the cirrhotic rats. Similar to minocycline, SB‐3CT did not affect splanchnic or hepatic vascular responsiveness to vasoconstrictors, or systemic vascular resistance and SMA resistance. As a result, the decreased SMA flow was less likely to be related to splanchnic vasoconstriction. Rather, the decreased mesenteric window vascular density detected by CD31 immunofluorescence indicated that the decreased SMA flow could be related to decreased splanchnic angiogenesis. This is supported by the downregulated expressions of mesenteric VEGF and phospho‐Erk protein in the SB‐3CT‐treated BDL cirrhotic rats.

Of note, both minocycline and SB‐3CT reduced CI in the BDL rats, whereas SB‐3CT did not affect CI in the sham rats. This suggests that MMP inhibition alleviates the systemic hyperdynamic circulatory condition characterized by high CI in cirrhosis. The reduced CI may be secondary to the reduced PP and alleviated portal hypertension. That is, MMP inhibition has the potential to improve hyperdynamic circulation by correcting portal hypertension.

To address more specifically the role of MMP‐9 in liver fibrosis and portal hypertension, we performed MMP‐9 KO mice experiments. Of note, BDL reduced MAP and elevated PP in both WT and MMP‐9 KO mice, indicating that BDL induced the systemic and portal hemodynamic features of portal hypertension in the mice. The MMP‐9 KO‐BDL mice had a significantly lower PP compared with the WT‐BDL mice. Since the KO‐BDL mice had a significantly lower Sirius red‐stained area than the WT‐BDL mice, the lower PP in the MMP‐9 KO mice may at least partially have been related to the amelioration of liver fibrosis. Consistently, the protein expression analyses of hepatic fibrogenesis factors revealed that BDL upregulated the protein expressions of hepatic α‐SMA (a marker of stellate cell activation) and phospho‐SMAD2 (a major factor in fibrogenesis) in the WT mice, but downregulated their expressions in the MMP‐9 KO mice.

An altered balance between MMPs and their inhibitors has been reported in experimental biliary fibrosis. MMPs have previously been suggested to be anti‐fibrotic factors, because they digest extracellular matrix (ECM) and ECM deposits in advanced fibrotic tissues. However, MMPs have recently been suggested to be profibrogenic mediators at the onset of fibrogenesis, especially MMP‐9. A previous study using an animal model of acute liver damage reported that interleukin‐1 induced MMPs through hepatic stellate cells within the space of Disse, followed by ECM degradation.^12^ This has been reported to lead to the collapse of sinusoids, leading to parenchymal cell death and the loss of liver function.^27^ Furthermore, type‐IV collagen in ECM, which is abundant in the space of Disse of normal livers, is a favoured substrate for MMP‐9. Breakdown of the naturally available ECM may allow quiescent hepatic stellate cells to activate or transdifferentiate. MMPs have also been shown to provoke the activation, migration and proliferation of hepatic stellate cells.^28^ Taken together, MMPs may act as “double‐edged swords” in the phases of tissue injury, early fibrogenesis, and advanced fibrosis. In the current study, we found that a nonselective MMP inhibitor, MMP‐2/MMP‐9 inhibitor and MMP‐9 gene deletion consistently attenuated liver fibrosis in the BDL animal model. These results indicate the potential of MMP‐9 inhibition in suppressing liver fibrosis.

Splanchnic hyperdynamic circulation has been demonstrated to be correlated with increased splenic blood flow, portal venous flow and porto‐systemic collateral circulation.^29^ Interestingly, terlipressin has been shown to decrease portal pressure and splenic blood flow at the same time,^30^ suggesting the participation of splenic blood flow in portal hypertension. In the current study, both minocycline and SB‐3CT significantly attenuated the severity of collateral shunting, extrahepatic angiogenesis and splanchnic blood flow. Therefore, the inhibition of MMP‐9 may ameliorate splenomegaly and reduce splenic blood flow while alleviating portal hypertension. Nevertheless, further clinical studies are warranted to elucidate this issue.

In conclusion, non‐specific MMP inhibition with minocycline and relatively specific MMP‐2/MMP‐9 inhibition with SB‐3CT ameliorated liver cirrhosis and portal hypertension‐related derangements. The beneficial findings of MMP‐9 inhibition were further strengthened by MMP‐9 gene deletion experiments. MMP‐9 inhibition is a possible target for the treatment of portal hypertension.

## CONFLICT OF INTEREST

All authors have no conflicts of interest to declare.

## AUTHOR CONTRIBUTIONS

**Hui‐Chun Huang:** Conceptualization (equal); Funding acquisition (lead); Writing‐review & editing (equal). **Hsin‐Ling Ho:** Investigation (lead); Methodology (equal). **Ching‐Chih Chang:** Supervision (equal). **Chiao‐Lin Chuang:** Supervision (equal). **Chon Kit Pun:** Investigation (equal); Writing‐original draft (lead). **Fa‐Yauh Lee:** Supervision (equal). **Yi‐Hsiang Huang:** Supervision (equal). **Ming‐Chih Hou:** Resources (equal). **Shao‐Jung Hsu:** Conceptualization (equal); Formal analysis (lead); Methodology (lead); Writing‐review & editing (lead).

## Supporting information

Fig S1‐2

Table S1

## Data Availability

The datasets used and analysed during the current study are available from the corresponding author on reasonable request.
