# Supplementary material for: Matrix metalloproteinase‐9 inhibition or deletion attenuates portal hypertension in rodents
Source: J Cell Mol Med. 2021 Oct 14;25(21):10073–87. doi: 10.1111/jcmm.16940 (PMC8572799; doi:10.1111/jcmm.16940)
Supplement: Supplementary file 1 — Fig S1‐2 [file JCMM-25-10073-s001.pdf]

Supplementary figure 1.

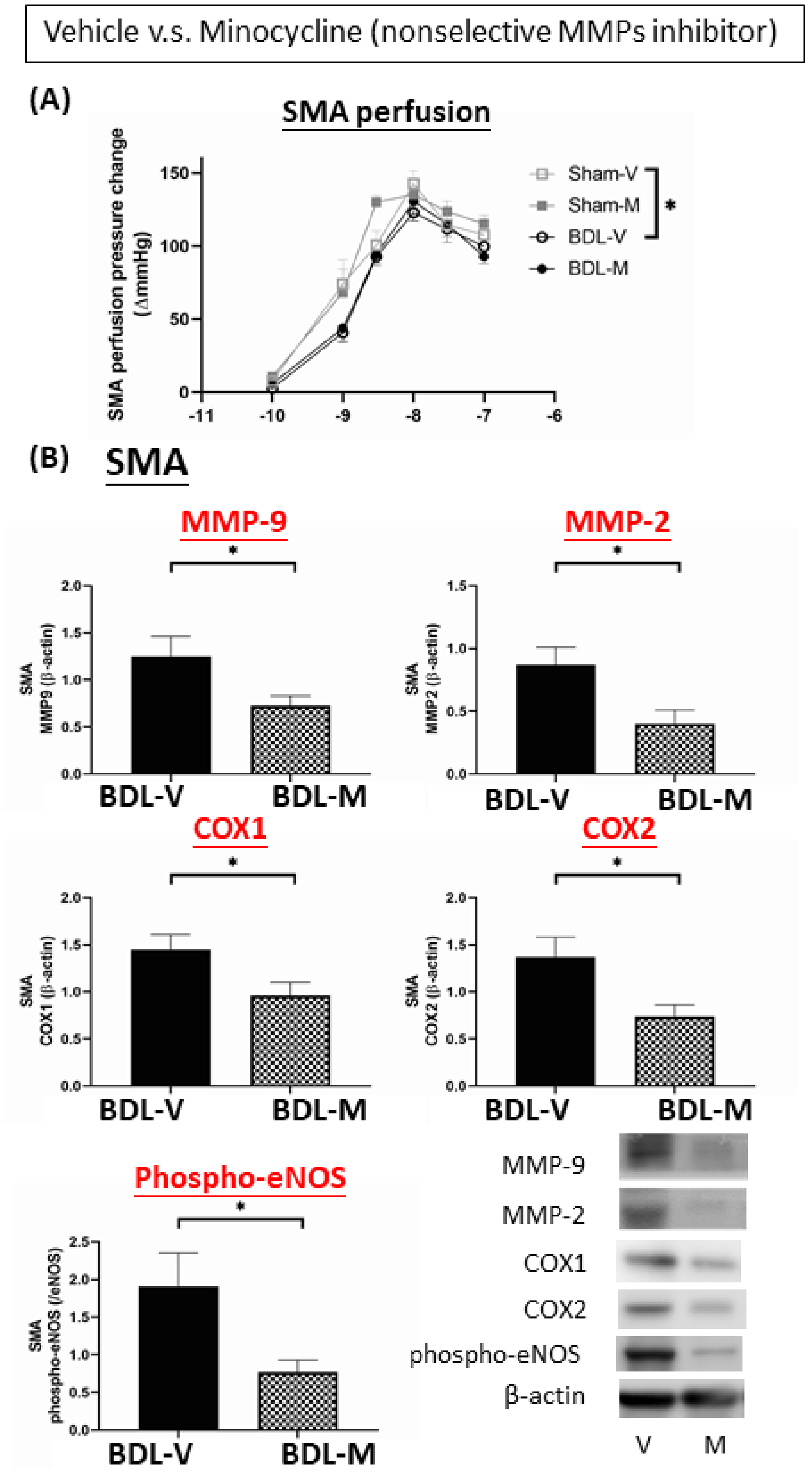

Supplementary figure 1. Effects of non-selective MMPs inhibitor minocycline on splanchnic vessels. (A) BDL rats had lower perfusion pressure changes to AVP while

comparing to sham rats in superior mesenteric artery (SMA) vascular territory. This suggested a poor vasoresponsiveness in splanchnic system of cirrhotic rats. Minocycline did not influence the splanchnic vascular contractility in cirrhotic rats. (B) Mesenteric angiogenesis factors protein expressions in BDL rats administered with vehicle or minocycline. Minocycline inhibited SMA MMP-9, MMP-2, COX1, COX2 and phospho-eNOS protein expressions in BDL rats. *$P<0.05$.

Supplementary figure 2.

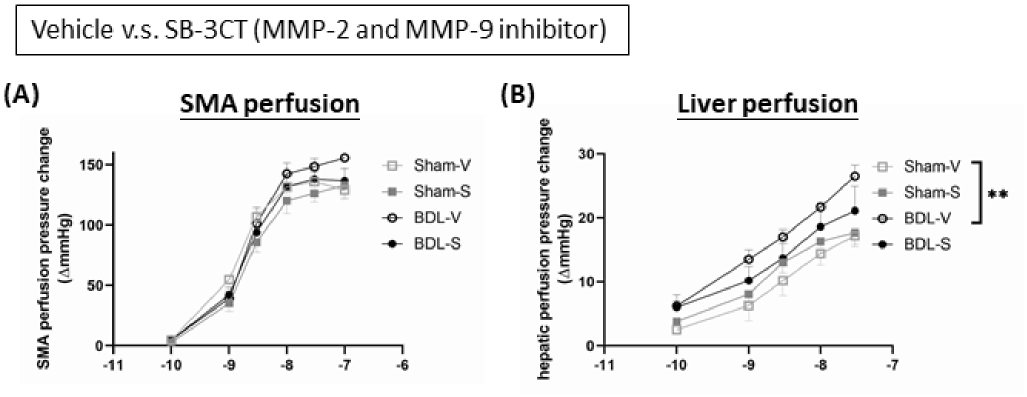

Supplementary figure 2. Effects of MMP-2/MMP-9 inhibitor SB-3CT on vascular responsiveness. (A) In both sham and BDL rats, the perfusion pressure changes to AVP were not significantly different between vehicle- and SB-3CT-treated groups. (B) SB-3CT did not affect hepatic vascular responsiveness to ET-1. Sham-V: sham-vehicle; sham-S: sham-SB-3CT. $^{**}P<0.01$.
