# Supplementary material for: Matrix metalloproteinase‐9 inhibition or deletion attenuates portal hypertension in rodents
Source: J Cell Mol Med. 2021 Oct 14;25(21):10073–87. doi: 10.1111/jcmm.16940 (PMC8572799; doi:10.1111/jcmm.16940)
Supplement: Supplementary file 2 — Table S1 [file JCMM-25-10073-s002.docx]

**Supplementary table 1. Plasma biochemistry parameters in wild type or MMP9 KO mice receiving sham or BDL operation**

|  |  | **Sham-WT** | **Sham-KO** | **BDL-WT** | **BDL-KO** |
| --- | --- | --- | --- | --- | --- |
|  |  | n=8 | n=8 | n=6 | n=5 |
| **Plasma biochemistry** | |  |  |  |  |
| ALT(U/L) | | 34±8 | 31±7 | 369±85§ | 278±37 |
| AST(U/L) | | 162±59 | 235±72 | 782±213§ | 870±147 |
| Total bilirubin  (mg/dl) | | <0.15 | <0.15 | 13.3±1.7§ | 12.9±1.3 |
| BUN(mg/dl) | | 49±11 | 45±6 | 57±7 | 47±3 |
| Creatinine(mg/dl) | | 0.13±0.03 | 0.16±0.01 | 0.17±0.00 | 0.17±0.00 |

WT: wild type; KO: MMP9 knockout; BDL: bile duct ligation; BW: body weight; ALT: alanine transaminase; BUN: blood urea nitrogen.

* P< 0.05, † P<0.01, ‡ P<0.001, WT groups compared to KO groups;

§ P<0.05, sham groups compared to BDL groups.
